# Novel therapeutic endoscope facilitates endoscopic submucosal dissection of adenocarcinoma at the esophagogastric junction

**DOI:** 10.1055/a-2316-3763

**Published:** 2024-06-05

**Authors:** Kohei Morioka, Yusaku Takatori, Teppei Masunaga, Naohisa Yahagi, Takanori Kanai, Motohiko Kato

**Affiliations:** 1 38084 Division of Research and Development for Minimally Invasive Treatment, Cancer Center, Keio University School of Medicine Graduate School of Medicine, Tokyo, Japan; 2 38084 Gastroenterology and Hepatology, Keio University School of Medicine Graduate School of Medicine, Tokyo, Japan; 3 38084 Center for Diagnostic and Therapeutic Endoscopy, Keio University School of Medicine Graduate School of Medicine, Tokyo, Japan

Endoscopic submucosal dissection (ESD) of lesions at the esophagogastric junction (EGJ) presents technical difficulties, with resection on the anal side presenting particular difficulties because of the steepness of the fornix 1 . Recently, a novel therapeutic endoscope has been reported as being useful for the treatment of lesions in the rectum and pharynx, organs with steep areas similar to those of the EGJ 2 3 . Here, we report an endoscopic resection performed using the novel endoscope for a lesion at the EGJ extending into the fornix ( Video 1 ).

An adenocarcinoma at the esophagogastric junction extending into the fornix is removed by endoscopic submucosal dissection using a novel thin therapeutic endoscope that facilitates the antegrade approach.Video 1

A 68-year-old woman was referred to our hospital for treatment of an adenocarcinoma at the EGJ. The lesion was 15 mm in diameter and the distal side of the lesion extended to the fornix ( Fig. 1 **a** ). We anticipated difficulties incising the fornix side of the lesion because the shallow downward angle of conventional therapeutic endoscopes hinders an antegrade approach. Furthermore, the steepness of the fornix can result in a perpendicular configuration between the endoscope and the fornix, even when using a retrograde approach. A novel thin therapeutic endoscope (EG-840TP; Fujifilm, Tokyo, Japan) has recently become commercially available in Japan, which has an outer diameter of 7.9 mm, a working channel diameter of 3.2  mm, and an adjustable downward angle of up to 160°. We initiated submucosal injection and a mucosal incision on the fornix side of the lesion; the downward angle of the endoscope enabled a smooth and successful antegrade approach. Next, we made a mucosal incision on the oral side of the lesion and continued the submucosal dissection without difficulties, still using only an antegrade approach ( Fig. 1 **b** ). Finally, we achieved en bloc resection without any adverse events ( Fig. 1 **c** and Fig. 2 ).

**Fig. 1:**
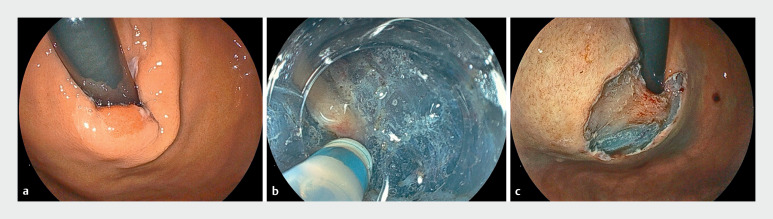
Endoscopic views showing: **a** an elevated lesion that is located at the esophagogastric junction, extending to the fornix; **b** endoscopic submucosal dissection (ESD) being carried out with an antegrade approach; **c** the post-ESD ulcer.

**Fig. 2:**
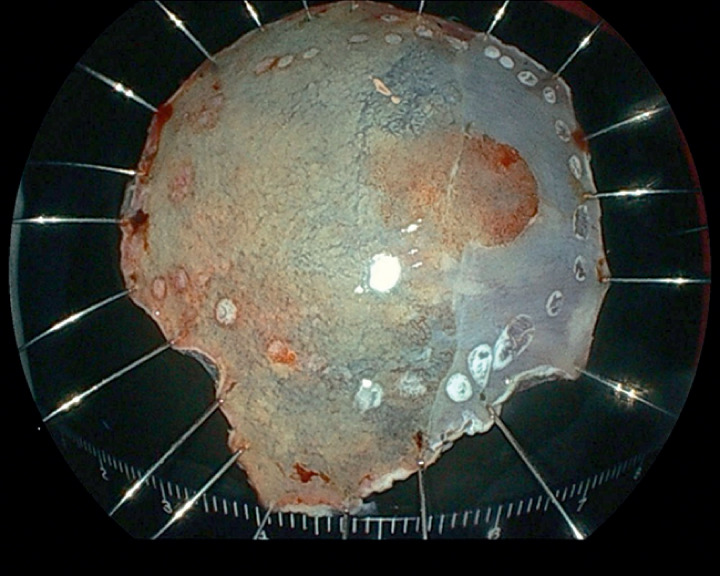
Photograph of the successfully excised en bloc resection specimen.

The pronounced downward angle of the novel endoscope resulted in an uncomplicated procedure, which was completed in 40 minutes. This novel endoscope may aid advancements in ESD technique, particularly in anatomically complex areas.

Endoscopy_UCTN_Code_TTT_1AO_2AG_3AD

## Notes

Endoscopy E-Videos https://eref.thieme.de/e-videos
*E-Videos* is an open access online section of the journal *Endoscopy* , reporting on interesting cases and new techniques in gastroenterological endoscopy. All papers include a high-quality video and are published with a Creative Commons CC-BY license. Endoscopy E-Videos qualify for HINARI discounts and waivers and eligibility is automatically checked during the submission process. We grant 100% waivers to articles whose corresponding authors are based in Group A countries and 50% waivers to those who are based in Group B countries as classified by Research4Life (see: https://www.research4life.org/access/eligibility/ ). This section has its own submission website at https://mc.manuscriptcentral.com/e-videos .

## References

[1] Kakushima N Yahagi N Fujishiro M Efficacy and safety of endoscopic submucosal dissection for tumors of the esophagogastric junction Endoscopy 2006 38 170 174 10.1055/s-2005-921039 16479425

[2] Takatori Y Matsuura N Nakayama A Endoscopic submucosal dissection using a novel therapeutic thin endoscope for a locally recurrent rectal tumor after endoluminal rectal surgery Endoscopy 2023 55 E1097 E1098 37802110 10.1055/a-2174-5398 PMC10558285

[3] Masunaga T Kato M Yahagi N Novel therapeutic thin endoscope facilitates endoscopic submucosal dissection for cervical esophageal cancer involving the pharyngoesophageal junction Endoscopy 2023 55 E602 E603 10.1055/a-2044-0512 37040889 PMC10089799

